# Tirzepatide after intensive lifestyle intervention in adults with overweight or obesity: the SURMOUNT-3 phase 3 trial

**DOI:** 10.1038/s41591-023-02597-w

**Published:** 2023-10-15

**Authors:** Thomas A. Wadden, Ariana M. Chao, Sriram Machineni, Robert Kushner, Jamy Ard, Gitanjali Srivastava, Bruno Halpern, Shuyu Zhang, Jiaxun Chen, Mathijs C. Bunck, Nadia N. Ahmad, Tammy Forrester

**Affiliations:** 1 Department of Psychiatry, Perelman School of Medicine at the University of Pennsylvania, Philadelphia, PA USA; 2 Johns Hopkins School of Nursing, Baltimore, MD USA; 3 Division of Endocrinology and Metabolism, Department of Medicine, Albert Einstein College of Medicine, Bronx, NY USA; 4 Department of Medicine, Northwestern University Feinberg School of Medicine, Chicago, IL USA; 5 Department of Epidemiology and Prevention, Wake Forest School of Medicine, Winston-Salem, NC USA; 6 Department of Medicine, Division of Diabetes, Endocrinology & Metabolism, Department of Pediatrics, Department of Surgery, Vanderbilt University School of Medicine, Nashville, TN USA; 7 Vanderbilt Weight Loss Clinics, Vanderbilt University Medical Center, Nashville, TN USA; 8 Obesity Group, Department of Endocrinology, Universidade de São Paulo, São Paulo, Brazil; 9 Eli Lilly and Company, Indianapolis, IN USA

**Keywords:** Obesity, Metabolic disorders

## Abstract

The effects of tirzepatide, a glucose-dependent insulinotropic polypeptide and glucagon-like peptide-1 receptor agonist, on weight reduction after successful intensive lifestyle intervention are unknown. This double-blind, placebo-controlled trial randomized (1:1) adults with body mass index ≥30 or ≥27 kg/m^2^ and at least one obesity-related complication (excluding diabetes), who achieved ≥5.0% weight reduction after a 12-week intensive lifestyle intervention, to tirzepatide maximum tolerated dose (10 or 15 mg) or placebo once weekly for 72 weeks (*n* = 579). The treatment regimen estimand assessed effects regardless of treatment adherence in the intention-to-treat population. The coprimary endpoint of additional mean per cent weight change from randomization to week 72 was met with changes of −18.4% (standard error (s.e.) 0.7) with tirzepatide and 2.5% (s.e. 1.0) with placebo (estimated treatment difference −20.8 percentage points (95% confidence interval (CI) −23.2%, −18.5%; *P* < 0.001). The coprimary endpoint of the percentage of participants achieving additional weight reduction ≥5% was met with 87.5% (s.e. 2.2) with tirzepatide and 16.5% (s.e. 3.0) with placebo achieving this threshold (odds ratio 34.6%; 95% CI 19.2%, 62.6%; *P* < 0.001). The most common adverse events with tirzepatide were gastrointestinal, with most being mild to moderate in severity. Tirzepatide provided substantial additional reduction in body weight in participants who had achieved ≥5.0% weight reduction with intensive lifestyle intervention. ClinicalTrials.gov registration: NCT04657016.

## Abstract (web summary)

In the SURMOUNT-3 trial, once-weekly treatment with tirzepatide was demonstrated to result in clinically meaningful additional weight loss in adults with overweight or obesity following initial successful weight loss of at least 5% body weight with intensive lifestyle intervention.

## Main

The adverse effects of obesity are well known to healthcare professionals and persons who live with this chronic disease^1–4^, as are the benefits of weight reduction. Decreasing baseline body weight by 5–10% reduces the likelihood of developing type 2 diabetes while also improving cardiometabolic risk factors (for example, blood pressure) and other obesity-related complications (for example, osteoarthritis)^5–8^.

Intensive lifestyle intervention is recommended as the cornerstone of obesity management^3,5,8–10^. It consists of a reduced-calorie diet (for example, 1,200–1,500 kcal per day based on weight or sex), physical activity (≥150 min per week) and frequent behavioral counseling (for example, ≥14 sessions over 6 months), and induces mean reductions of 5–8% of baseline weight with accompanying improvements in health^5^. Its overall effectiveness, however, is limited by two factors. Large weight reductions are critical for achieving optimal control of obesity-related complications (for example, obstructive sleep apnea and nonalcoholic steatohepatitis)^6–8^ and decreasing cardiovascular mortality^11–14^, but <20% of patients treated with lifestyle interventions lose ≥15% of baseline weight^15^. Patients also regain one-third of lost weight in the year following treatment, with increasing weight regain over time^5,16^. Weight regain after diet and exercise intervention is attributable, in part, to persistent metabolic adaptations in which patients’ hunger hormones increase, satiety hormones decrease and energy expenditure declines out of proportion to the amount of weight lost^17–19^ such that an even lower energy intake is needed to maintain the weight-reduced state.

New incretin-based, antiobesity medications could bolster the results of intensive lifestyle intervention^20^. Semaglutide 2.4 mg is a glucagon-like peptide-1 (GLP-1) receptor agonist, originally approved at a lower dose for control of type 2 diabetes and which, in persons with obesity or overweight (but not diabetes), reduces baseline body weight by 15% at up to 2 years (versus 2–3% for placebo)^21,22^. It decreases energy intake principally by modification of hunger and satiety signaling in select neural regions^21^. Tirzepatide is a single molecule that combines glucose-dependent insulinotropic polypeptide and GLP-1 receptor agonism^23^ to exert synergistic effects on appetite (for example, hunger and satiety), energy intake and metabolic function^24–26^. It is approved in many geographies including the USA, European Union and Japan as a once-weekly subcutaneous injectable for type 2 diabetes and is currently under review for chronic weight management^26–28^. In the SURMOUNT-1 trial, patients with obesity or overweight (but not diabetes) who received tirzepatide 15 mg, with monthly brief lifestyle counseling, lost 20.9% of baseline weight at 72 weeks (versus 3.1% for placebo) with accompanying reductions in cardiometabolic risk factors^27^.

Expert panels have suggested the use of antiobesity medications following intensive lifestyle intervention to induce additional weight reduction (which may be needed to achieve optimal control of obesity-related complications) or, at a minimum, to prevent weight regain^5,7,9,10^. The present trial evaluated the efficacy of tirzepatide at 72 weeks postrandomization in adults with obesity or overweight (but not diabetes) who successfully lost ≥5% of baseline weight during a 12-week lead-in period that provided intensive lifestyle intervention.

## Results

### Patient disposition

#### Intensive lifestyle intervention lead-in period

**Fig. 1:**
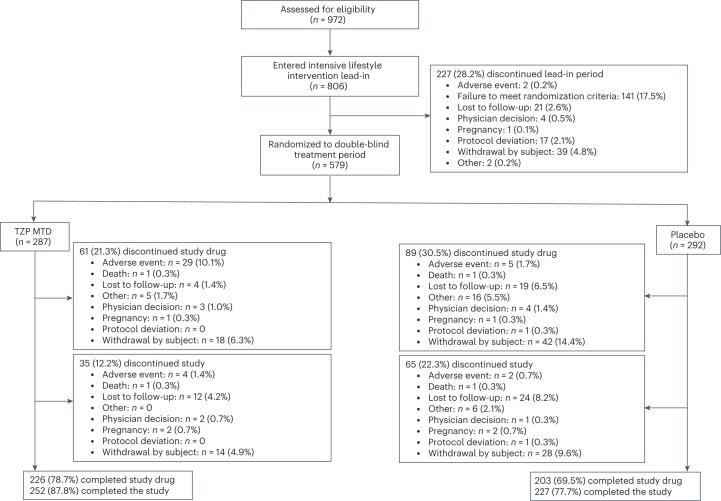
Trial profile. SURMOUNT-3 CONSORT flow diagram. MTD, maximum tolerated dose (10 or 15 mg). TZP, tirzepatide.

A total of 972 participants were assessed for eligibility at screening, of whom 806 were enrolled into the 12-week intensive lifestyle intervention lead-in period (Fig. 1). The first participant was enrolled on 12 April 2021 and the last on 3 September 2021. The key demographics and clinical characteristics of these participants have previously been published^28^.

Of the 806 participants enrolled, 579 (71.8%) who achieved ≥5% weight reduction at the end of the lead-in period and were otherwise eligible to proceed to the next phase of the study were randomized to either tirzepatide maximum tolerated dose (MTD, *n* = 287) or placebo (*n* = 292) (Fig. 1). Mean body weight and body mass index (BMI) in these 579 participants decreased from 109.5 kg and 38.6 kg/m^2^, respectively, at screening to 101.9 kg and 35.9 kg/m^2^, respectively, at randomization, representing an average 6.9% reduction in body weight after the 12-week intensive lifestyle intervention (Table 1). Weight reduction during lead-in was accompanied by reductions in waist circumference, systolic and diastolic blood pressure, glycated hemoglobin A_1c_ (HbA_1c_), fasting glucose and fasting insulin. There were mean improvements in all lipid levels, except for high-density lipoprotein (HDL) cholesterol and free fatty acids (Table 1).

**Table 1:** Clinical characteristics and changes during intensive lifestyle intervention lead-in period

	Mean (s.d.)								
	Tirzepatide MTD (*n* = 287)			Placebo (*n* = 292)			Total (*n* = 579)		
	Start of intensive lifestyle intervention lead-in	Start of double-blind treatment period (randomization)	Change during lead-in	Start of intensive lifestyle intervention lead-in	Start of double-blind treatment period (randomization)	Change during lead-in	Start of intensive lifestyle intervention lead-in	Start of double-blind treatment period (randomization)	Change during lead-in
Body weight, kg	110.1 (23.9)	102.5 (22.1)	−7.6 (2.9) kg −6.9 (1.9)%	108.9 (22.2)	101.3 (20.7)	−7.6 (2.8) kg −7.0 (2.0)%	109.5 (23.0)	101.9 (21.4)	−7.6 (2.9) kg −6.9 (2.0)%
BMI, kg/m^2^	38.7 (6.6)	36.1 (6.1)	−2.7 (0.9)	38.4 (6.8)	35.7 (6.4)	−2.7 (0.9)	38.6 (6.7)	35.9 (6.3)	−2.7 (0.9)
Waist circumference, cm	115.9 (15.6)	109.3 (15.2)	−6.6 (5.4)	116.3 (15.3)	109.6 (15.1)	−6.7 (4.9)	116.1 (15.4)	109.4 (15.0)	−6.7 (5.2)
Blood pressure, mmHg									
Systolic	125.9 (12.7)	121.4 (12.7)	−4.5 (11.4)	126.0 (13.3)	120.5 (12.4)	−5.4 (11.3)	126.0 (13.0)	121.0 (12.6)	−5.0 (11.4)
Diastolic	81.8 (8.5)	79.1 (8.9)	−2.6 (8.1)	81.2 (8.4)	78.1 (9.2)	−3.1 (8.2)	81.5 (8.5)	78.6 (9.1)	−2.9 (8.1)
Pulse rate, beats per min	73.4 (10.0)	72.0 (10.8)	−1.4 (10.2)	72.2 (9.9)	70.4 (10.3)	−1.8 (9.1)	72.8 (9.9)	71.2 (10.6)	−1.6 (9.6)
HbA_1c_, %	5.5 (0.4)	5.3 (0.4)	−0.1 (0.3)	5.5 (0.4)	5.4 (0.4)	−0.1 (0.3)	5.5 (0.4)	5.4 (0.4)	−0.1 (0.3)
Fasting glucose, mg dl^−^^1^	95.7 (9.9)	92.6 (11.3)	−3.1 (10.1)	94.0 (8.8)	91.3 (9.4)	−2.8 (10.0)	94.9 (9.4)	91.9 (10.4)	−2.9 (10.0)
Fasting insulin, mIU l^−1^	97.7 (75.3)	70.7 (59.0)	−18.5 (52.9)%	93.6 (87.7)	62.9 (44.4)	−22.3 (41.3)%	95.6 (81.7)	66.7 (52.2)	−20.4 (47.4)%
Lipid level, mg dl^−^^1^									
Total cholesterol	191.4 (36.8)	185.2 (37.2)	−2.5 (13.7)%	196.2 (39.0)	185.3 (38.2)	−4.9 (12.1)%	193.8 (38.0)	185.3 (37.6)	−3.7 (13.0)%
Non-HDL cholesterol	141.9 (35.8)	136.7 (35.6)	−2.3 (18.1)%	145.6 (37.5)	135.90 (35.7)	−5.5 (15.4)%	143.7 (36.7)	136.3 (35.6)	−3.9 (16.8)%
HDL cholesterol	49.6 (14.0)	48.4 (12.7)	−0.8 (13.9)%	50.6 (13.8)	49.3 (12.9)	−1.5 (13.4)%	50.1 (13.9)	48.9 (12.8)	−1.2 (13.7)%
LDL cholesterol	113.7 (30.4)	112.5 (32.5)	0.8 (24.3)%	118.0 (32.4)	112.3 (32.3)	−3.6 (18.2)%	115.9 (31.5)	112.4 (32.4)	−1.4 (21.5)%
VLDL cholesterol	60.3 (27.2)	54.4 (21.7)	−3.8 (33.1)%	62.1 (30.9)	54.2 (24.4)	−5.5 (34.3)%	61.2 (29.1)	54.3 (23.1)	−4.7 (33.7)%
Triglycerides	141.2 (112.3)	121.4 (55.7)	−4.4 (33.4)%	138.2 (73.5)	118.6 (53.3)	−6.0 (34.1)%	139.7 (94.7)	120.0 (54.5)	−5.2 (33.8)%
Free fatty acids, mEq l^−^^1^	0.6 (0.2)	0.6 (0.3)	23.0 (82.0)%	0.5 (0.2)	0.6 (0.2)	29.9 (86.3)%	0.5 (0.2)	0.6 (0.2)	26.5 (84.2)%
eGFR, ml min^−^^1^ 1.73 m^−^^2^	99.0 (17.1)	95.6 (17.1)	−3.4 (10.4)	100.3 (15.7)	97.1 (16.7)	−3.3 (8.9)	99.6 (16.4)	96.4 (16.9)	−3.3 (9.7)
Patient-reported outcomes									
SF-36v2 physical functioning domain score^a^	48.9 (7.8)	51.7 (6.7)	2.7 (7.7)	48.6 (7.8)	51.7 (6.8)	3.1 (5.8)	48.8 (7.8)	51.7 (6.7)	2.9 (6.8)
IWQOL-Lite-CT physical function composite score^b^	59.5 (22.7)	73.4 (21.3)	13.9 (17.6)	57.4 (24.3)	71.4 (22.0)	13.9 (17.7)	58.4 (23.5)	72.4 (21.6)	13.9 (17.7)

eGFR, estimated glomerular filtration rate; HDL-C, high-density lipoprotein cholesterol; IWQOL-Lite-CT, Impact of Weight on Quality of Life-Lite-Clinical Trials Version; LDL-C, low-density lipoprotein cholesterol; SF-36v2, Short Form-36v.2 Health Survey acute form; VLDL-C, very-low-density lipoprotein cholesterol.

^a^SF-36v2 measures health-related quality of life and general health status. SF-36v2 scores are norm based—that is, scores are transformed to a scale in which the 2009 US general population has a mean score of 50 and s.d. of 10. An increase in score represents an improvement in health status.

^b^IWQOL-Lite-CT measures weight-specific, health-related quality of life. All items are rated on either a five-point frequency scale (‘never’ to ‘always’) or a five-point truth scale (‘not at all true’ to ‘completely true’). Scores are transformed to a scale of 0–100, with higher scores reflecting better levels of functioning.

#### Tirzepatide versus placebo postrandomization

The majority of the 579 randomized participants were white (86.0%) and female (62.9%), with an overall mean age of 45.6 years (Table 2). The average duration of obesity was 15.1 years, and 66.1% had a medical history of one or more obesity-related complications. Demographics and clinical characteristics at randomization (week 0), as well as weight reduction and cardiometabolic changes during the lead-in period, were similar across the tirzepatide MTD (10 or 15 mg) and placebo groups (Tables 1 and 2 and Extended Data Table 1).

**Table 2:** Baseline characteristics (at randomization) in all randomized participants

Characteristics	Tirzepatide MTD (*n* = 287)	Placebo (*n* = 292)	Total (*n* = 579)
Age, mean (s.d.), years	45.4 (12.6)	45.7 (11.8)	45.6 (12.2)
Sex, no. (%)			
Female	181 (63.1)	183 (62.7)	364 (62.9)
Male	106 (36.9)	109 (37.3)	215 (37.1)
Race, no. (%)^a^			
Asian	2 (0.7)	2 (0.7)	4 (0.7)
Black or African American	31 (10.8)	32 (11.0)	63 (10.9)
Multiple	6 (2.1)	2 (0.7)	8 (1.4)
American Indian or Alaskan	2 (0.7)	4 (1.4)	6 (1.0)
White	246 (85.7)	252 (86.3)	498 (86.0)
Ethnicity, no. (%)^a^			
Hispanic or Latino	151 (52.6)	161 (55.1)	312 (53.9)
Not Hispanic or Latino	132 (46.0)	129 (44.2)	261 (45.1)
Not reported	4 (1.4)	2 (0.7)	6 (1.0)
Country			
Argentina	43 (15.0)	44 (15.1)	87 (15.0)
Brazil	59 (20.6)	60 (20.5)	119 (20.6)
USA	185 (64.5)	188 (64.4)	373 (64.4)
Duration of obesity, mean (s.d.), years^b^	15.4 (11.6)	14.8 (10.8)	15.1 (11.2)
BMI category, no. (%)			
<27	5 (1.7)	12 (4.1)	17 (2.9)
≥27 to <30	32 (11.1)	38 (13.0)	70 (12.1)
≥30 to <35	100 (34.8)	107 (36.6)	207 (35.8)
≥35 to <40	95 (33.1)	79 (27.1)	174 (30.1)
≥40	55 (19.2)	56 (19.2)	111 (19.2)
Obesity-related complications, *n* (%)^c^			
Hypertension	95 (33.1)	104 (35.6)	199 (34.4)
Dyslipidemia	71 (24.7)	81 (27.7)	152 (26.3)
ASCVD	5 (1.7)	6 (2.1)	11 (1.9)
Polycystic ovarian syndrome	8 (4.4)	8 (4.4)	16 (4.4)
Obstructive sleep apnea	25 (8.7)	34 (11.6)	59 (10.2)
Osteoarthritis	43 (15.0)	48 (16.4)	91 (15.7)
Anxiety/depression	61 (21.3)	55 (18.8)	116 (20.0)
NAFLD	9 (3.1)	16 (5.5)	25 (4.3)
Asthma or COPD	21 (7.3)	31 (10.6)	52 (9.0)
Gout	6 (2.1)	9 (3.1)	15 (2.6)
Number of weight-related complications, *n* (%)^c^			
0	96 (33.4)	100 (34.2)	196 (33.9)
1	102 (35.5)	81 (27.7)	183 (31.6)
2	48 (16.7)	54 (18.5)	102 (17.6)
3	22 (7.7)	36 (12.3)	58 (10.0)
4	14 (4.9)	14 (4.8)	28 (4.8)
≥5	5 (1.7)	7 (2.4)	12 (2.1)

ASCVD, atherosclerotic cardiovascular disease; COPD, chronic obstructive pulmonary disease; NAFLD, non-alcoholic fatty liver disease.

^a^Race and ethnicity were determined by the participant according to fixed selection categories.

^b^Duration of obesity was assessed by self-report.

^c^Baseline medical conditions were assessed through a review of participants’ medical history.

Of the 579 randomized participants, 479 (82.7%) completed the study (87.8% on tirzepatide MTD and 77.7% on placebo) and 429 (74.1%) completed the study on treatment (78.7% on tirzepatide MTD and 69.5% on placebo). The most common reasons for discontinuation of study treatment were adverse event (10.5%, detailed in Table 3) and withdrawal by subject (6.3%) in the tirzepatide MTD group, and withdrawal by subject (14.4%) and lost to follow-up (6.5%) in the placebo group.

**Table 3:** Adverse events during the double-blind period and safety follow-up period (safety analysis set)

	No. (%)	
	Tirzepatide MTD (*n* = 287)	Placebo (*n* = 292)
Participants with ≥1 adverse event	250 (87.1)	224 (76.7)
Serious adverse events	17 (5.9)	14 (4.8)
Death^a^	1 (0.3)	1 (0.3)
Adverse events leading to treatment discontinuation^b^	30 (10.5)	6 (2.1)
Nausea	24 (8.4)	4 (1.4)
Vomiting	6 (2.1)	0
Diarrhea	3 (1.0)	0
Dyspepsia	3 (1.0)	0
Constipation	2 (0.7)	0
Adverse events occurring in ≥5% of participants in any treatment group		
Nausea	114 (39.7)	41 (14.0)
Diarrhea	89 (31.0)	27 (9.2)
Constipation	66 (23.0)	20 (6.8)
COVID-19	66 (23.0)	74 (25.3)
Vomiting	52 (18.1)	4 (1.4)
Injection site reaction	32 (11.1)	3 (1.0)
Abdominal pain	30 (10.5)	7 (2.4)
Decreased appetite	27 (9.4)	12 (4.1)
Dyspepsia	27 (9.4)	9 (3.1)
Headache	27 (9.4)	22 (7.5)
Upper respiratory tract infection	25 (8.7)	21 (7.2)
Alopecia	20 (7.0)	4 (1.4)
Dizziness	20 (7.0)	6 (2.1)
Fatigue	20 (7.0)	9 (3.1)
Flatulence	19 (6.6)	8 (2.7)
Gastroesophageal reflux disease	19 (6.6)	7 (2.4)
Back pain	17 (5.9)	15 (5.1)
Eructation	16 (5.6)	3 (1.0)
Influenza	12 (4.2)	25 (8.6)
Urinary tract infection	11 (3.8)	15 (5.1)
Anxiety	9 (3.1)	19 (6.5)
Arthralgia	7 (2.4)	15 (5.1)
Sinusitis	6 (2.1)	16 (5.5)
Adverse events of special interest		
Severe or serious gastrointestinal events	16 (5.6)	5 (1.7)
Malignancies	5 (1.7)	3 (1.0)
Severe or serious acute gall bladder diseases	2 (0.7)	0
MACE (adjudication confirmed)	1 (0.3)	1 (0.3)
Pancreatitis (adjudication confirmed)	1 (0.3)	1 (0.3)
Severe or serious renal events	1 (0.3)	0
Severe or serious MDD/suicidal behavior and ideation	1 (0.3)	0
Severe or serious arrhythmias and cardiac conduction disorders	0	1 (0.3)
Severe hypoglycemia	0	0
Other adverse events of interest		
Cholelithiasis	4 (1.4)	3 (1.0)
Acute cholecystitis	1 (0.3)	0
Chronic cholecystitis	0	1 (0.3)

Events are listed according to Medical Dictionary for Regulatory Activities, v.26.0, preferred terms. MACE, major adverse cardiovascular event; MDD, major depressive disorder.

^a^Deaths are also included as serious adverse events and discontinuations due to adverse event.

^b^Only adverse events occurring in ≥2 participants in any treatment group are presented.

In tirzepatide-treated participants, 248 (86.4%) had a tirzepatide MTD of 15 mg. In this study, all randomized participants took at least one dose of the study intervention (tirzepatide MTD or placebo). Therefore, the intention-to-treat population is the same as the modified intention-to-treat population.

### Primary outcomes

**Fig. 2:**
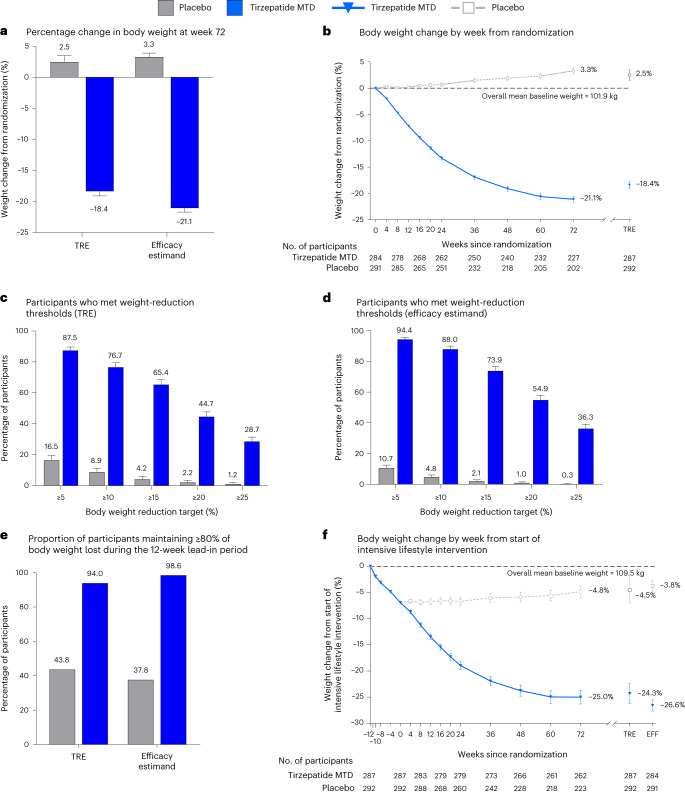
Effect of once-weekly tirzepatide on body weight in comparison with placebo. **a**, Least-square mean (LSM) (s.e.) per cent change in body weight from randomization to week 72 derived from an analysis of covariance model for the TRE (tirzepatide MTD, *n* = 287 participants; placebo, *n* = 292 participants), and from MMRM analysis for the efficacy estimand (tirzepatide MTD, *n* = 284 participants; placebo, *n* = 291 participants). **b**, LSM (s.e.) per cent change in body weight over time from randomization to 72 weeks, derived from MMRM analysis for the efficacy estimand; week 72 estimates for the TRE are also shown. **c**,**d**, LSM (s.e.) percentages of participants who had body weight reduction of at least 5, 10, 15, 20 or 25% from randomization to week 72. **c**, Percentage of participants reaching weight reduction thresholds (TRE) was calculated using logistic regression with missing values imputed by hybrid imputation (tirzepatide MTD, *n* = 287 participants; placebo; *n* = 292 participants). **d**, Percentage of participants reaching weight reduction thresholds (efficacy estimand) was obtained by logistic regression with missing values at week 72 imputed from MMRM analysis (tirzepatide MTD, *n* = 284 participants; placebo, *n* = 291 participants). **e**, LSM proportion of participants that maintained ≥80% of body weight reductions achieved at the end of the lead-in period. Both TRE and efficacy estimand shown. **f**, Mean (95% CI) per cent change in body weight over time from the start of the intensive lifestyle intervention lead-in period (–12 weeks) to 72 weeks, derived from observed values, irrespective of treatment adherence; week 72 estimates for TRE and efficacy estimand (EFF), are also shown.

**Extended Data Fig. 1:**
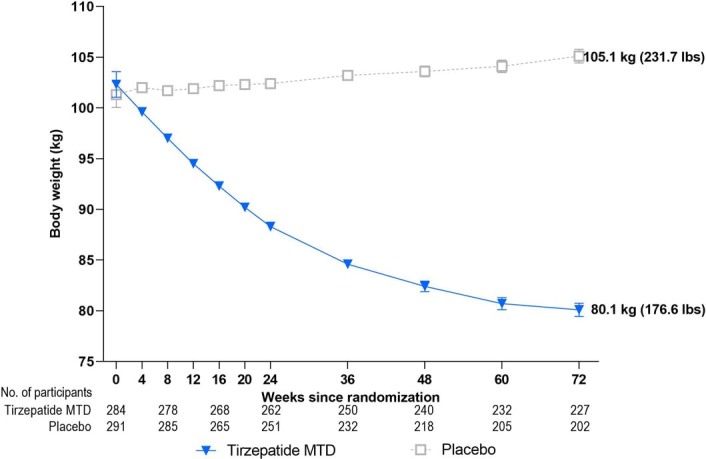
Body weight in kg over time. Mean (standard error) body weight (kg) over time from randomization to 72 weeks derived from a mixed-model for repeated measures (MMRM) analysis for the efficacy estimand. Only participants with non-missing baseline value and at least one non-missing post-baseline value of the response variable were included in analysis. MTD, maximum tolerated dose (10 or 15 mg).

Figure 2a,b shows the mean percentage reduction in body weight from randomization to week 72. For the treatment regimen estimand (TRE) the mean change at week 72 was −18.4% (s.e. 0.7) with tirzepatide MTD and 2.5% (s.e. 1.0) with placebo. Tirzepatide MTD was superior to placebo, with an estimated treatment difference relative to placebo of −20.8 percentage points (95% CI −23.2, −18.5; *P* < 0.001) (Table 4). The mean change in body weight for the efficacy estimand was −21.1% (s.e. 0.6) with tirzepatide MTD and 3.3% (s.e. 0.6) with placebo. The estimated treatment difference was −24.5 percentage points (95% CI −26.1, −22.8; *P* < 0.001) for tirzepatide MTD versus placebo. Absolute body weight over time is shown in Extended Data Fig. 1.

**Table 4:** Efficacy findings from randomization (week 0) to week 72

	LSM (s.e.)		Treatment comparison	
	Tirzepatide MTD (*n* = 287)	Placebo (*n* = 292)	Difference from placebo (95% CI)	*P* value
**Primary endpoints**				
Per cent change in body weight	−18.4 (0.7)	2.5 (1.0)	ETD −20.8 (−23.2, −18.5)	<0.001
Participants achieving ≥5% body weight reduction, %	87.5 (2.2)	16.5 (3.0)	OR 34.6 (19.2, 62.6)	<0.001
**Key secondary endpoints**				
Participants achieving body weight reduction, %				
≥10%	76.7 (2.7)	8.9 (2.4)	OR 34.7 (17.6, 68.3)	<0.001
≥15%	65.4 (3.0)	4.2 (1.8)	OR 48.2 (19.2, 121.0)	<0.001
≥20%	44.7 (3.0)	2.2 (1.3)	OR 40.4 (12.2, 133.8)	<0.001
Participants maintaining ≥80% of lead-in body weight lost at week 72, %	94.0 (1.7)	43.8 (3.9)	ETD 19.7 (10.3, 37.6)	<0.001
Change in waist circumference, cm	−14.6 (0.7)	0.2 (1.0)	ETD −14.8 (−17.2, −12.5)	<0.001
**Additional secondary endpoints**				
Change in body weight, kg	−21.5 (0.7)	3.5 (0.7)	ETD −25.0 (−26.9, −23.2)	NR^a^
Change in BMI, kg/m^2^	−7.7 (0.2)	1.2 (0.2)	ETD −8.9 (−9.6, −8.3)	NR
Change in SBP, mmHg	−5.1 (0.7)	4.1 (0.7)	ETD −9.2 (−11.2, −7.2)	NR
Change in DBP, mmHg	−3.2 (0.5)	2.3 (0.5)	ETD –5.5 (−6.9, −4.1)	NR
Fasting lipids				
Per cent change in total cholesterol	−3.0 (1.0)	5.2 (1.1)	ETD −7.8 (−10.4, −5.1)	NR
Per cent change in non-HDL cholesterol	−9.8 (1.3)	5.6 (1.5)	ETD −14.6 (−17.9, −11.2)	NR
Per cent change in HDL cholesterol	15.4 (1.2)	3.6 (1.1)	ETD 11.4 (8.2, 14.7)	NR
Per cent change in LDL cholesterol	−6.1 (1.4)	6.1 (1.7)	ETD −11.5 (−15.3, −7.5)	NR
Per cent change in VLDL cholesterol	−25.6 (1.6)	3.0 (2.3)	ETD −27.8 (−32.1, −23.2)	NR
Per cent change in triglycerides	−25.8 (1.6)	3.0 (2.3)	ETD −28.0 (−32.3, −23.4)	NR
Per cent change in free fatty acids	−33.1 (2.2)	−15.0 (3.0)	ETD −21.3 (−28.4, −13.6)	NR
Change in fasting glucose, mg dl^−1^	−8.8 (0.8)	2.4 (0.9)	ETD −11.2 (−13.5, −8.8)	NR
Change in HbA1c, %	−0.5 (0.0)	0.0 (0.0)	ETD −0.5 (−0.5, −0.4)	NR
Per cent change in fasting insulin	−39.1 (2.5)	17.3 (5.0)	ETD −48.1 (−53.7, −41.7)	NR
Patient-reported outcomes				
Change in SF-36v2 Physical Functioning domain score^b^	3.3 (0.4)	−0.6 (0.4)	ETD 3.9 (2.8, 4.9)	NR
Change in IWQOL-Lite-CT Physical Function composite score^c^	13.9 (1.1)	1.1 (1.2)	ETD 12.8 (9.7, 16.0)	NR
**Prespecified exploratory endpoints**				
Participants achieving body weight reduction ≥25%, %	28.7 (2.7)	1.2 (0.9)	OR 33.70 (8.84, 128.52)	NR

Primary, key secondary and prespecified exploratory endpoints are presented using the TRE, and additional secondary endpoints are presented using the efficacy estimand. Primary and key secondary endpoints were controlled for type 1 error at a two-sided significance level of 0.05 within each estimand via a graphical testing approach. Other endpoints were not controlled for type 1 error.

DBP, diastolic blood pressure; ETD, estimated treatment difference; NR, not reported; SBP, systolic blood pressure.

^a^*P* values are not reported for additional secondary and prespecified exploratory endpoints because these were not controlled for type 1 error.

^b^SF-36v2 measures health-related quality of life and general health status. SF-36v2 scores are norm based—that is, transformed to a scale in which the 2009 US general population has a mean score of 50 and s.d. of 10. An increase in score represents an improvement in health status.

^c^IWQOL-Lite-CT measures weight-specific, health-related quality of life. All items are rated on either a five-point frequency scale (‘never’ to ‘always’) or a five-point truth scale (‘not at all true’ to ‘completely true’). Scores are transformed to a scale of 0–100, with higher scores reflecting better levels of functioning.

For the TRE, 87.5% (251) of participants in the tirzepatide MTD group lost an additional ≥5% of body weight from randomization to week 72 compared with 16.5% (48) in the placebo group (odds ratio (OR) 34.6 (95% CI 19.2, 62.6); *P* < 0.001) (Fig. 2c and Table 4). For the efficacy estimand, 94.4% (268) of participants in the tirzepatide MTD group had an additional body weight reduction of ≥5% from randomization compared with 10.7% (31) in the placebo group (OR 130.4 (95% CI 70.0, 242.8); *P* < 0.001) (Fig. 2d).

### Secondary outcomes

#### Change in body weight

At week 72, more participants on tirzepatide MTD than placebo achieved reductions in body weight of ≥10, ≥15 and ≥20% from randomization (*P* < 0.001; Fig. 2c,d and Table 4).

At 72 weeks, for the TRE, 94.0% (270) of participants in the tirzepatide MTD group maintained ≥80% of body weight lost during the 12-week lead-in period compared with 43.8% (128) in the placebo group (OR 19.7; 95% CI 10.3, 37.6; *P* < 0.001; Fig. 2e and Table 4). For the efficacy estimand, 98.6% (280) of participants in the tirzepatide MTD group met this endpoint compared with 37.8% (110) in the placebo group (OR 101.6; 95% CI 39.2, 263.6; *P* < 0.001; Fig. 2e).

Overall, for the TRE, intensive lifestyle intervention followed by 72 weeks of tirzepatide led to a total weight change of −24.3% compared with −4.5% with intensive lifestyle intervention followed by placebo (estimated treatment difference −19.9 percentage points (95% CI −23.5, −16.2) (Fig. 2f). For the efficacy estimand, intensive lifestyle intervention followed by 72 weeks of tirzepatide led to a total weight change of –26.6% compared with −3.8% with intensive lifestyle intervention followed by placebo (estimated treatment difference −22.8 percentage points (95% CI −24.3, −21.2; Fig. 2f and Extended Data Table 2).

Accordingly, there was a reduction in BMI with tirzepatide compared with placebo from randomization to week 72 (efficacy estimand: tirzepatide, −7.7 kg/m^2^ versus placebo, 1.2 kg/m^2^; estimated treatment difference −8.9 kg/m^2^ (95% CI −9.6, −8.3; Table 4). Total change in BMI with intensive lifestyle intervention followed by 72 weeks of tirzepatide MTD was −10.4 kg/m^2^ compared with –1.4 kg/m^2^ with intensive lifestyle intervention followed by placebo (efficacy estimand: estimated treatment difference −8.9 kg/m^2^ (95% CI −9.6, −8.3; Extended Data Table 2).

#### Cardiometabolic risk factors and physical function

**Extended Data Fig. 2:**
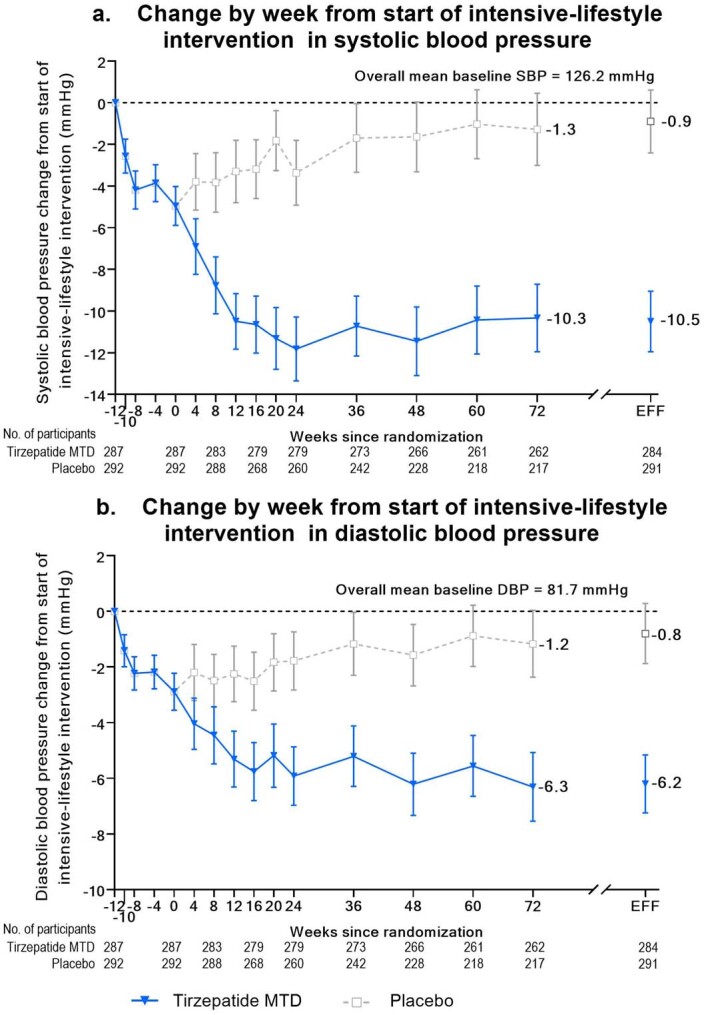
Blood pressure change from start of lead-in period over time. Panel A, mean (95% confidence interval) change from baseline over time in systolic blood pressure from start of intensive-lifestyle intervention lead-in period (week -12) to 72 weeks using observed means. Week 72 estimates for the efficacy estimand (EFF) are also shown. Panel B, mean (95% confidence interval) change from baseline over time in diastolic blood pressure from start of intensive-lifestyle intervention lead-in period (week -12) to 72 weeks using observed means. Week 72 estimates for the efficacy estimand (EFF) are also shown.

At week 72 the change from randomization in waist circumference with tirzepatide MTD was superior to placebo using the TRE (tirzepatide, −14.6 cm versus placebo, 0.2 cm; estimated treatment difference, −14.8 cm (95% CI −17.2, −12.5; *P* < 0.001; Table 4). Results were consistent for the efficacy estimand (tirzepatide, −16.8 cm versus placebo, 1.1 cm; estimated treatment difference −17.9 cm (95% CI −19.5, −16.3; *P* < 0.001). Improvements with tirzepatide MTD, from randomization to week 72, were greater versus placebo in both systolic blood pressure (tirzepatide, −5.1 mmHg versus placebo, 4.1 mmHg; estimated treatment difference −9.2 mmHg (−11.2, −7.2) and diastolic blood pressure (tirzepatide, −3.2 mmHg versus placebo, 2.3 mmHg; estimated treatment difference −5.5 mmHg (−6.9, −4.1) using the efficacy estimand (Table 4 and Extended Data Fig. 2). Treatment with tirzepatide MTD resulted in further improvements across all fasting lipid levels (HDL, LDL, VLDL, total cholesterol, triglycerides and free fatty acids), glycemic control (fasting glucose and HbA1c) and fasting insulin compared with placebo at 72 weeks from randomization (Table 4). In addition, 4.9 and 2.8% of participants in the tirzepatide group compared with 1.0 and 1.7% of participants in the placebo group were reported as having a decrease in intensity of antihypertensive and lipid-lowering medications, respectively. Conversely, 2.4 and 0.3% of participants in the tirzepatide group were reported to have experienced an increase in intensity of antihypertensive and lipid-lowering therapies, respectively, compared with 6.5 and 2.1% of participants in the placebo group.

Participant-reported physical function improved more with tirzepatide than with placebo from randomization to week 72 (Table 4). This was observed with both the physical functioning domain score for Short Form-36v.2 Health Survey (SF-36v2) (tirzepatide, 3.3 versus placebo, −0.6; estimated treatment difference 3.9 (95% CI 2.8, 4.9)) and the Impact of Weight on Quality of Life-Lite-Clinical Trials Version (IWQOL-Lite-CT) physical function composite score (tirzepatide, 13.9 versus placebo, 1.1; estimated treatment difference 12.8 (95% CI 9.7, 16.0)) using the efficacy estimand.

Changes in cardiometabolic parameters and patient-reported outcomes from the start of the lead-in period (week −12) to week 72 are reported in Extended Data Table 2.

### Safety

**Extended Data Fig. 3:**
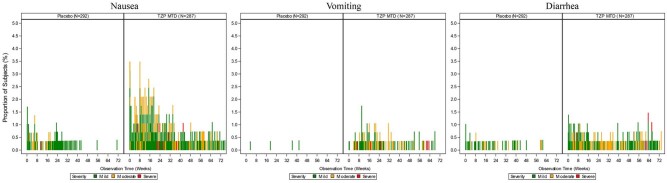
Incidence of nausea, vomiting, and diarrhea over time. The percentage of participants receiving tirzepatide or placebo who reported nausea, vomiting, or diarrhea are presented. Percentages are based on number of participants at risk at specific observation time. Events were classed as mild (shown in green), moderate (shown in orange), or severe (shown in red). MTD, maximum tolerated dose (10 or 15 mg); TZP, tirzepatide.

Overall, 87.1% of the 287 tirzepatide-treated participants reported at least one treatment-emergent adverse event compared with 76.7% of the 292 placebo-treated participants (Table 3). The most frequently reported adverse events were gastrointestinal (nausea, diarrhea and constipation). These occurred in more participants in the tirzepatide MTD group than placebo, were mostly mild to moderate in severity and occurred primarily during dose escalation (Extended Data Fig. 3). Antiemetic medication use was reported by 78 participants (27.2%) treated with tirzepatide and by 20 (6.8%) treated with placebo. Antidiarrheal medication use was reported by 23 participants (8.0%) treated with tirzepatide and by six (2.1%) treated with placebo.

Serious adverse events were reported by 31 participants (5.4%) overall. Occurrence was similar in participants treated with tirzepatide (5.9%) and placebo (4.8%) (Table 3). Two deaths (both myocardial infarction) were reported during the study, one in the tirzepatide MTD group and one in the placebo group. Both events were considered not to be related to the study treatment by the investigator.

Adjudication-confirmed cases of pancreatitis were reported in 0.3% (one) of participants in the tirzepatide MTD group and 0.3% (one) of participants in the placebo group from randomization to safety follow-up (Table 3). Cholelithiasis was reported in 1.4% (four) of participants in the tirzepatide group and 1.0% (three) of participants in the placebo group. There was one case (0.3%) of acute cholecystitis in the tirzepatide group and none in the placebo group. Malignancies were reported in 1.7% (five) of participants in the tirzepatide MTD group and 1.0% (three) of participants in the placebo group. None of the malignancies were considered related to the study treatment by the investigators, and no cases of medullary thyroid cancer or pancreatic cancer were reported. Additional safety measures are reported in Extended Data Table 3.

### Exploratory outcomes

For the prespecified exploratory endpoint of achieving ≥25% body weight reduction from randomization, for the TRE, 28.7% (82) of tirzepatide-treated participants compared with 1.2% (four) in the placebo group met this target (OR 33.7 (95% CI 8.8, 128.5); Table 4). Results were consistent for the efficacy estimand (tirzepatide, 36.3% versus placebo, 0.3%; OR 124.6 (95% CI 24.9, 623.2); Fig. 2d).

## Discussion

Tirzepatide substantially increased the magnitude of weight loss when administered following an initial 12-week intensive lifestyle intervention that reduced baseline body weight by an average of 6.9% in successful program completers. As measured from randomization (week 0) to week 72, participants who received tirzepatide MTD of 10 or 15 mg lost an additional 18.4% of body weight, compared with a gain of 2.5% for placebo. In total, 87.5% of tirzepatide-treated participants lost an additional 5% or more of their randomization weight compared with 16.5% of placebo-treated participants, with tirzepatide also demonstrating superiority in the achievement of all other categorical weight losses. These findings indicate that individuals with overweight or obesity who have lost approximately 5–10% of their body weight with supervised lifestyle intervention—or potentially through their own self-directed diet and exercise efforts—could expect to achieve further clinically meaningful weight loss with the addition of tirzepatide.

The strength of tirzepatide is underscored by comparison with a similarly designed trial of liraglutide (3.0 mg), approved for chronic weight management. After losing an average 6.0% of baseline weight in a comparable lead-in program, participants who received liraglutide achieved an additional 6.2% reduction in randomization weight at 56 weeks compared with a 0.2% reduction for placebo^29^. The liraglutide trial provided a total of 17 lifestyle counseling sessions during the medication phase of the study compared with only quarterly visits in the present trial. This decreased frequency of counseling visits could explain the greater weight regain in the placebo group in the present study. The only other similarly designed trial of a medication approved for chronic weight management found that orlistat (120 mg three times daily) was not effective in inducing additional weight loss over 1 year when administered following an average 11.0% reduction achieved with intensive lifestyle intervention^30^. Patients treated by both orlistat and placebo regained one-third or more of their lost weight. Tirzepatide was also superior to placebo on a traditional measure of weight loss maintenance—the proportion of participants who maintained a predefined percentage of their initial weight loss. In the present study, 94% of tirzepatide-treated participants, compared with 44% of those who received placebo, maintained ≥80% of their weight loss achieved in the lead-in period. These results compare favorably with those achieved with both liraglutide and orlistat but perhaps, more importantly, with the results of traditional lifestyle intervention. Individuals who receive such intervention typically regain one-third of their lost weight in the year following treatment completion^31^. Regain can be decreased to 10–15% at 1 year with participation in a weight loss maintenance program, offered in person or by phone, which provides continued lifestyle counseling on a monthly or more frequent basis^32^. However, after 2.5 years of such monthly phone-based counseling only 45% of participants maintained ≥4 kg of an original mean 8.5 kg loss achieved during a 6 month lead-in period^33^. These findings reveal the potential benefits of tirzepatide, relative to traditional weight loss maintenance counseling, in not only sustaining weight reduction achieved with intensive lifestyle intervention but in adding to it. Long-term comparative studies for weight loss maintenance are needed.

The cumulative 24.3% reduction in body weight achieved with intensive lifestyle intervention, followed by tirzepatide, approximates the 1 year weight loss induced with sleeve gastrectomy^34^. The overall BMI reduction of 10 kg/m^2^ represents a downward shift of about two BMI categories. Participants treated with lifestyle interventions have long sought to achieve a similar magnitude of weight loss, principally to improve their health and quality of life^35,36^. Tirzepatide enhanced the improvements in cardiometabolic risk factors that were achieved in the lead-in period. Systolic and diastolic BP improved by an additional −5.1 and −3.2 mmHg, respectively, lipids parameters improved by an additional −3% to −26% and fasting insulin further declined by 39%. Self-reported physical function improved by 3.3 points on the SF-36v2 physical functioning domain score and by 13.9 points on the IWQOL-Lite-CT physical function composite. These improvements underscore the additional benefits that patients may receive from treatment with tirzepatide after first losing weight with intensive lifestyle intervention, or potentially with their own self-directed diet and activity programs. By contrast, many of the cardiometabolic improvements achieved during the lead-in reverted toward baseline in the placebo group.

The safety profile of tirzepatide in this trial was consistent with findings from previous trials of tirzepatide when evaluated for the treatment of obesity^27,37^ or type 2 diabetes^38^, as well as with the safety profile of the GLP-1 receptor agonist class in patients with obesity or overweight^39,40^. Mild-to-moderate gastrointestinal events were the most frequent treatment-emergent adverse events, mostly transient and occurring during dose escalation. Compared with the tirzepatide 15 mg group in SURMOUNT-1, the tirzepatide group in this study had modestly higher rates of gastrointestinal adverse events and treatment discontinuation due to adverse events. Other trials that combined intensive lifestyle intervention with pharmacotherapy have also shown higher rates of gastrointestinal events compared with trials investigating pharmacotherapy without intensive lifestyle intervention (for example, STEP-3 compared with STEP-1 for semaglutide 2.4 mg and SCALE-MAINTENANCE compared with SCALE for liraglutide 3.0 mg)^21,29,41,42^. It has been speculated that caloric restriction could lead to a reduction in GLP-1 and other gastrointestinal satiety hormones^43^. Whether this worsens initial gastrointestinal tolerability to incretin-based therapy and is a possible explanation for the observed findings requires further investigation.

Much remains to be learned about how lifestyle intervention and the new incretin-based antiobesity medications can be optimally used together. If the goal of combining these therapies is to increase total weight loss, results of the present trial and SCALE-MAINTENANCE^29^ suggest that introducing the intensive lifestyle intervention first (for approximately 12 weeks) followed by the addition of medication, as in the present study, could maximize weight reduction. The weight reduction observed with tirzepatide MTD in the 72 week, double-blind period of the current trial was similar to that achieved with tirzepatide 10 and 15 mg over 72 weeks in the SURMOUNT-1 study. Therefore, the sequential use of these interventions appeared to produce additive weight loss that approached the combined results of each intervention used alone. However, providing intensive lifestyle intervention and medication concurrently, rather than sequentially, has not achieved the same degree of additive benefit in placebo-controlled trials^41,44^. For example, the concurrent provision of intensive lifestyle intervention (plus meal replacements) and semaglutide 2.4 mg in the STEP-3 trial produced a mean weight loss of 16.0%, which was comparable to that observed in the STEP-1 study (14.9%) in a similar population that did not receive this enhanced intensive lifestyle intervention^21,41^.

The suggestion of additivity with sequential therapy, however, may be challenged by findings from preclinical studies. These studies have demonstrated that caloric restriction alone does not address the underlying physiology regulating body weight or fat mass, and antiobesity medication has the same overall ultimate effect regardless of whether or not caloric restriction preceded the medication^45^. This implies that, if weight reduction in the lead-in period of the present trial was due only to a volitional reduction in caloric intake, the overall weight reduction of 24.3% could represent the effect the drug would have had even without an intensive lifestyle lead-in. Indeed, a recent 88 week trial of tirzepatide has demonstrated this degree of weight reduction as early as 52 weeks on treatment^46^. The reason the weight reduction in the present trial may be higher than that observed in SURMOUNT-1 could be related to differences in demographics between the trial populations, or to the fact that this study, and other similarly designed trials, preselected for a highly responsive population by randomization of only participants who achieved an initial reduction of 5% or more with intensive lifestyle intervention. It is possible that participants who respond to lifestyle intervention are simply more responsive to tirzepatide. Further analyses should help examine this hypothesis.

Another major treatment issue concerns the intensity (that is, frequency) and scope of lifestyle intervention required with antiobesity medications. Weekly lifestyle visits and daily monitoring of food and energy intake historically have been required to help patients achieve and maintain the 500–750 kcal per day deficit needed to induce clinically meaningful weight loss^16^. Semaglutide and tirzepatide both appear to physiologically drive this reduction in energy intake, which might enable patients to implement dietary behavior changes with greater ease and efficiency than conventional lifestyle counseling. Similarly, weight loss induced with antiobesity medication, with the accompanying improvement in physical function observed in the present study, could increase patients’ ease in engaging in physical activity, thus potentially further improving their cardiometabolic health.

The strengths of this study, which included an intensive lifestyle lead-in, include the fact that it had a relatively large sample size in which over one-third of the randomized population were men and over half were of Hispanic ethnicity. In addition, a 72-week treatment period facilitated at least 52 weeks of treatment with tirzepatide at MTD. The allowance of dose de- and re-escalation during the titration phase helped to maximize tolerability and reflected dose adjustment strategies that may be relevant for clinical practice.

The study’s limitations include that it was geographically restricted to North and South America and that the study population was predominantly white, thus potentially limiting the generalizability of the findings. In addition, the 17.5% of participants who did not lose at least 5% of baseline weight in the intensive lifestyle intervention were not randomized to medication. To the extent that response to lifestyle intervention may predict response to medication, exclusion of these participants may have resulted in a higher mean weight loss with tirzepatide MTD than would have been observed if lifestyle nonresponders had been included. Trials of the response to antiobesity medications in persons who are unsuccessful with intensive lifestyle intervention are needed, because lack of success with lifestyle interventions has been a common prerequisite for initiation of pharmacotherapy or bariatric surgery. Future studies evaluating both genetic and behavioral predictors of response to lifestyle intervention and pharmacotherapy will help inform clinical management even earlier in the course of treatment.

In conclusion, in the SURMOUNT-3 trial, tirzepatide demonstrated clinically meaningful additional body weight reductions in adults with overweight or obesity following initial weight loss with intensive lifestyle intervention.

## Methods

### Study design and participants

**Extended Data Fig. 4:**
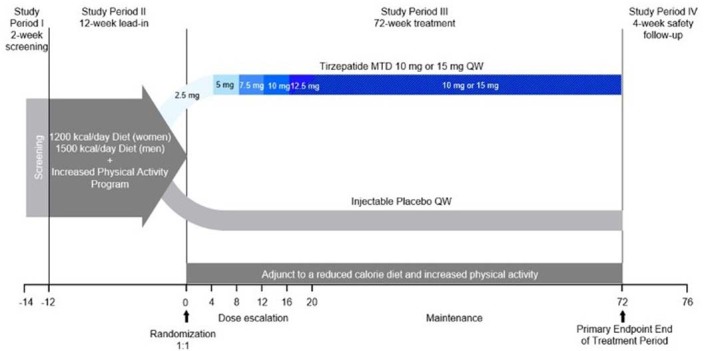
SURMOUNT-3 study design. This is a phase 3, multicenter, randomized, placebo-controlled, double- blinded clinical trial investigating the efficacy and safety of maximum tolerated dose (MTD) of tirzepatide (10 mg or 15 mg) administered once weekly (QW) subcutaneously compared with placebo for body weight management, in participants who have obesity, or overweight with at least 1 obesity-related complication (excluding type 2 diabetes), and with at least 5% weight reduction following a 12-week intensive lifestyle intervention lead-in. All randomized participants were planned to undergo a 72-week treatment period.

This 84-week, multicenter, randomized, parallel-arm, double-blind, placebo-controlled trial was conducted at 62 medical research centers in the USA, Argentina and Brazil. The study consisted of four periods: a 2-week screening period; a 12-week lead-in period during which participants received intensive lifestyle intervention to achieve ≥5.0% body weight reduction; a 72-week double-blind, placebo-controlled treatment period (including a 20-week dose escalation period); and a 4-week safety follow-up period (Extended Data Fig. 4).

Eligible participants were ≥18 years of age and had obesity (BMI ≥ 30 kg/m^2^) or overweight (BMI ≥ 27 kg/m^2^) with at least one weight-related complication. Female enrollment was capped at 70% to ensure adequate representation of the male population. Full eligibility criteria are listed below.

### Inclusion criteria

Participants were eligible for inclusion in the study only if all of the following criteria applied:

#### Type of participant and disease characteristics

1. had a BMI of:
  ≥30 kg/m^2^ or
  ≥27 kg/m^2^ and previously diagnosed with at least one of the following weight-related comorbidities:
    hypertension: treated or with systolic blood pressure ≥130 mmHg or diastolic blood pressure ≥80 mmHg
    dyslipidemia: treated or with LDL ≥ 160 mg dl^−^^1^ (4.1 mmol l^−^^1^) or triglycerides ≥150 mg dl^−^^1^ (1.7 mmol l^−^^1^) or HDL < 40 mg dl^−^^1^ (1.0 mmol l^−^^1^) for men, or HDL < 50 mg dl^−^^1^ (1.3 mmol l^−^^1^) for women
    obstructive sleep apnea
    cardiovascular disease (for example, ischemic cardiovascular disease, New York Heart Association Functional Classification Class I–III heart failure)
2. had a history of at least one self-reported unsuccessful dietary effort to lose body weight
3. in the investigator’s opinion, were well motivated, capable and willing to:
  learn how to self-inject study drug, as required for this protocol (visually impaired persons who were not able to perform the injections must have had the assistance of a sighted individual trained to inject the study drug; persons with physical limitations who were not able to perform the injections must have had the assistance of an individual trained to inject the study drug)
  inject study drug (or receive an injection from a trained individual if visually impaired or with physical limitations)
  follow study procedures for the duration of the study, including—but not limited to—following lifestyle advice (for example, dietary restrictions, exercise plan), maintaining a study diary and completing required questionnaires

#### Participant characteristics

4. were at least 18 years of age and age of majority according to local laws and regulations
  - male participants:
    Male participants with partners of childbearing potential should have been willing to use reliable contraceptive methods throughout the study and for five half-lives of study drug plus 90 days, corresponding to 4 months after the last injection.
  - female participants:
    Female participants not of childbearing potential may have participated and included those who were:
      infertile due to surgical sterilization (hysterectomy, bilateral oophorectomy or tubal ligation) or congenital anomaly (such as Mullerian agenesis) or
      postmenopausal—defined as either:
        a woman at least 40 years of age with an intact uterus, not on hormone therapy and who had cessation of menses for at least 1 year without an alternative medical cause, and follicle-stimulating hormone ≥40 mIU ml^−^^1^; women in this category must have tested negative in pregnancy test before study entry
          or
        a woman 55 years or older not on hormone therapy and who had at least 12 months of spontaneous amenorrhea
          or
        a woman at least 55 years of age with a diagnosis of menopause before starting hormone replacement therapy
    Female participants of childbearing potential (not surgically sterilized and between menarche and 1 year postmenopausal) must have:
      tested negative for pregnancy at visit 1 based on a serum pregnancy test
      if sexually active, agreed to use two forms of effective contraception where at least one form was highly effective for the duration of the trial plus 30 days, corresponding to 2 months after the last injection; and
      not have been breastfeeding

Note: contraceptive use by men or women should have been consistent with local regulations regarding the methods of contraception for those participating in clinical studies.

#### Informed consent

5. Participants were required to be capable of giving signed informed consent, which included compliance with the requirements and restrictions listed in the informed consent form and in this protocol.

### Exclusion criteria

Participants were excluded from study enrollment if they met any of the following criteria at screening:

#### Medical conditions

##### *Diabetes related*

6. had type 1 or type 2 diabetes mellitus, history of ketoacidosis or hyperosmolar state/coma
7. had at least one laboratory value suggestive of diabetes mellitus during screening, including one or more of: HbA1c ≥6.5% (≥48 mmol mol^−^^1^), fasting glucose ≥126 mg dl^−^^1^ (≥7.0 mmol l^−^^1^) or random glucose ≥200 mg dl^−^^1^ (≥11.1 mmol l^−^^1^)

##### *Obesity related*

8. had a self-reported change in body weight >5 kg within 3 months before screening
9. had a previous planned surgical treatment for obesity (excluding liposuction or abdominoplasty, if performed >1 year before screening)
10. had or planned to have endoscopic and/or device-based therapy for obesity or had device removal within the past 6 months before screening:
  mucosal ablation
  gastric artery embolization
  intragastric balloon
  duodenal–jejunal endoluminal liner

##### *Other medical*

11. had renal impairment measured as eGFR < 30 ml min^−^^1^ 1.73 m^−^^2^, calculated by Chronic Kidney Disease-Epidemiology as determined by central laboratory during screening
12. had a known clinically important gastric emptying abnormality (for example, severe gastroparesis or gastric outlet obstruction) or chronically took drugs that directly affect GI motility
13. had a history of chronic or acute pancreatitis
14. had thyroid-stimulating hormone (TSH) outside of the range 0.4–6.0 mIU l^−^^1^ at the screening visit
  Note: participants receiving treatment for hypothyroidism may have been included, provided their thyroid hormone replacement dose had been stable for at least 3 months and their TSH at screening fell within the range indicated above.
  Note: participants with a history of subclinical hypothyroidism but a TSH at screening within the range indicated above may have been included if, in the investigator’s opinion, the patient was unlikely to require initiation of thyroid hormone replacement during the course of the study.
15. had obesity induced by other endocrinologic disorders (for example, Cushing syndrome) or diagnosed monogenetic or syndromic forms of obesity (for example, melanocortin 4 receptor deficiency or Prader–Willi syndrome)
16. had a history of substantial active or unstable major depressive disorder (MDD) or other severe psychiatric disorder (for example, schizophrenia, bipolar disorder or other serious mood or anxiety disorder) within the past 2 years
  Note: participants with MDD or generalized anxiety disorder and whose disease state was considered stable for the past 2 years and was expected to remain stable throughout the course of the study, in the opinion of the investigator, may have been considered for inclusion if they were not on excluded medications
17. had a lifetime history of suicide attempt
18. had a PHQ-9 score of 15 or more at visit 1
19. on the Columbia Suicide Severity Rating Scale (C-SSRS) at any time from visit 1 to visit 2:
  a ‘yes’ answer to Question 4 (active suicidal ideation with some intent to act, without specific plan) on the ‘Suicidal Ideation’ portion of the C-SSRS
    or
  a ‘yes’ answer to Question 5 (active suicidal ideation with specific plan and intent) on the ‘Suicidal Ideation’ portion of the C-SSRS
    or
  a ‘yes’ answer to any of the suicide-related behaviors (actual attempt, interrupted attempt, aborted attempt, preparatory act or behavior) on the ‘Suicidal Behavior’ portion of the C-SSRS
    and
  the ideation or behavior occurred within the past month
20. had uncontrolled hypertension (systolic blood pressure ≥160 mmHg and/or diastolic blood pressure ≥100 mmHg)
21. had any of the following cardiovascular conditions within 3 months before visit 2:
  acute myocardial infarction
  cerebrovascular accident (stroke)
  unstable angina
  hospitalization due to congestive heart failure
22. had New York Heart Association Functional Classification Class IV congestive heart failure
23. had acute or chronic hepatitis, signs and symptoms of any other liver disease other than nonalcoholic fatty liver disease (NAFLD) or any of the following, as determined by the central laboratory during screening:
  alanine aminotransferase level >3.0 times upper limit of normal (ULN) for the reference range
  alkaline phosphatase level >1.5 times ULN for the reference range
  total bilirubin level >1.2 times ULN for the reference range (except for cases of known Gilbert syndrome)
  Note: participants with NAFLD were eligible to participate in this trial if their alanine aminotransferase level was ≤3.0 times ULN for the reference range.
24. had a serum calcitonin level (at visit 1) of
  ≥20 ng l^−^^1^, if eGFR ≥60 ml min^−^^1^ 1.73 m^−^^2^
  ≥35 ng l^−^^1^, if eGFR <60 ml min^−^^1^ 1.73 m^−^^2^
25. had a family or personal history of medullary thyroid carcinoma or multiple endocrine neoplasia syndrome type 2
26. had a history of an active or untreated malignancy or were in remission from a clinically important malignancy (other than basal or squamous cell skin cancer, in situ carcinomas of the cervix or in situ prostate cancer) for <5 years
27. had any other condition not listed in this section (for example, hypersensitivity or intolerance) that is a contraindication to GLP-1 R agonists
28. had a history of any other condition (such as known drug or alcohol abuse, diagnosed eating disorder or other psychiatric disorder) that, in the opinion of the investigator, may have precluded the participant from following and completing the protocol
29. had a history of use of marijuana or tetrahydrocannabinol-containing products within 3 months of enrollment, or unwillingness to abstain from marijuana or tetrahydrocannabinol-containing products use during the trial
  Note: if a participant had used cannabidiol oil during the past 3 months but agreed to refrain from use for the duration of the study, the participant could be enrolled.
30. had had a transplanted organ (corneal transplants (keratoplasty) were allowed) or were awaiting an organ transplant
31. had any hematological condition that may have interfered with HbA1c measurement (for example, hemolytic anemias, sickle cell disease)

#### Previous and/or concomitant therapy

32. were receiving or had received within 3 months before screening chronic (>2 weeks or 14 days) systemic glucocorticoid therapy (excluding topical, intraocular, intranasal, intra-articular or inhaled preparations) or had evidence of a substantial, active autoimmune abnormality (for example, lupus or rheumatoid arthritis) that had required (within the past 3 months) or was likely to require, in the opinion of the investigator, concurrent treatment with systemic glucocorticoids (excluding topical, intraocular, intranasal, intra-articular or inhaled preparations) during the course of the study
33. had current treatment with or history of (within 3 months before visit 2) treatment with medications that may cause substantial weight gain, including but not limited to: tricyclic antidepressants, atypical antipsychotics and mood stabilizers
  Examples:
  imipramine
  amitriptyline
  mirtazapine
  paroxetine
  phenelzine
  chlorpromazine
  thioridazine
  clozapine
  olanzapine
  valproic acid (and its derivatives) or
  lithium
  Note: selective serotonin reuptake inhibitors other than paroxetine were permitted.
34. had taken, within 3 months before visit 2, medications (prescribed or over-the-counter) or alternative remedies that promote weight loss

Examples included, but were not limited to

- Saxenda (liraglutide 3.0 mg)
- Xenical/Alli (orlistat)
- Meridia (sibutramine)
- Acutrim (phenylpropanolamine)
- Sanorex (mazindol)
- Apidex (phentermine)
- BELVIQ (lorcaserin)
- Bontril (phendimetrazine)
- Qsymia (phentermine/topiramate combination)
- Contrave (naltrexone/bupropion)

Note: use of metformin, or any other glucose-lowering medication, whether prescribed for polycystic ovarian syndrome or diabetes prevention, was not permitted.

35. had started implantable or injectable contraceptives (such as Depo Provera) within 18 months before screening

#### Previous and/or concurrent clinical study experience

36. were currently enrolled in any other clinical study involving an investigational product or any other type of medical research judged not to be scientifically or medically compatible with this study
37. within the past 30 days had participated in a clinical study and received treatment, whether active or placebo. If the study involved an investigational product, five half-lives or 30 days, whichever was longer, should have passed.
38. had previously completed or withdrawn from this study or any other study investigating tirzepatide after receiving at least one dose of investigational product

#### Other exclusions

39. were investigator site personnel directly affiliated with this study and/or their immediate families. Immediate family was defined as a spouse, parent, child or sibling, whether biological or legally adopted.
40. were Lilly employees

This study is registered with ClinicalTrials.gov, NCT04657016. The protocol was approved by local institutional review boards and the trial complied with the International Conference on Harmonization Good Clinical Practice guidelines and the Declaration of Helsinki. All participants provided written informed consent.

### Lead-in period

Eligible participants were enrolled in a 12-week intensive lifestyle intervention lead-in period. The lead-in lifestyle intervention included frequent in-person lifestyle counseling sessions (that is, eight sessions over 12 weeks), delivered by a dietitian or similarly qualified healthcare professional. Women were instructed to consume approximately 1,200 kcal per day and men 1,500 kcal per day. The dietary intervention could include up to two meal replacements (liquid meal replacements or prepackaged, portion-controlled meals) per day. Participants were encouraged to engage in at least 150 min of moderate-intensity physical activity per week (for example, brisk walking). They were counseled on behavior modification strategies to help implement and adhere to the diet and exercise recommendations, and were encouraged to complete 3-day diet and exercise logs before each counseling visit.

### Randomization for the double-blind treatment period

Participants who achieved ≥5.0% weight reduction at the end of the 12-week lead-in period were randomly assigned in a 1:1 ratio to receive either the MTD of tirzepatide (10 or 15 mg) or placebo. Assignment to treatment group was determined by a computer-generated random sequence using a validated interactive web-response system. All participants, investigators and the sponsor were masked to treatment assignment. To maintain masking of participants and site staff, the single-dose pens were identical between active product and placebo. Randomization was stratified according to country, sex (female, male) and per cent weight reduction at the end of lead-in (<10 versus ≥10%).

### Procedures during the double-blind treatment period

Tirzepatide and matched placebo were administered once weekly as a subcutaneous injection using a single-dose pen. The starting dose of tirzepatide was 2.5 mg, increasing by 2.5 mg every 4 weeks until an MTD dose of 10 or 15 mg was reached. To optimize tolerability and adherence, gastrointestinal symptoms could be managed by dietary counseling, symptomatic medications according to the investigator’s discretion or skipping of a single dose of treatment. During the first 24 weeks of the treatment period, if these mitigations were not successful one cycle of tirzepatide dose de- and re-escalation (in 2.5 mg increments) was allowed for participants unable to tolerate any dose between 7.5 and 15 mg inclusive; participants unable to tolerate 2.5 or 5 mg were discontinued from study drug but remained in the study for continued follow-up. Participants who did not tolerate up to 10 mg even after one de- and re-escalation attempt were discontinued from study drug but remained in the study for continued follow-up. Dose adjustments were not permitted after the first 24 weeks of treatment.

Throughout the postrandomization period, participants continued to consult with a dietitian or other qualified healthcare professional. Lifestyle counseling sessions occurred every 12 weeks and focused on consumption of a healthy balanced diet, with a 500 kcal per day deficit and continuation of physical activity. Use of the diet and exercise log was encouraged. In between counseling sessions, diet and exercise goals were reinforced by site staff at every monthly visit.

Participants were permitted to use concomitant medications that they required during the study, except for certain agents specified in the protocol that could interfere with the assessment of efficacy and safety characteristics of the study treatments.

### Study outcomes

Coprimary endpoints were per cent change in body weight and the proportion of study participants who achieved ≥5% weight reduction from randomization to week 72. Key secondary endpoints, controlled for type 1 error rate, included the proportion of study participants who achieved ≥10, ≥15 or ≥20% weight reduction from randomization to week 72. The proportion of study participants who achieved ≥25% reduction in body weight was a prespecified exploratory endpoint. Key secondary endpoints also included the proportion of participants who, at week 72, maintained ≥80% of the body weight loss achieved during the 12-week lead-in period, as well as change in waist circumference (cm) from randomization to week 72.

Additional secondary endpoints included change in anthropometrics (absolute body weight and BMI), cardiometabolic risk factors (blood pressure, lipids, fasting glucose, HbA_1c_ and fasting insulin) and patient-reported outcomes (the Physical Functioning domain score on the SF-36v2 acute form, and the IWQOL-Lite-CT Physical Function composite score). These additional secondary endpoints were evaluated both from randomization (week 0) and from the start of the lead-in period (week –12) to week 72.

In addition, changes in the intensity of antihypertensive and lipid-lowering therapies in the double-blind period, as reported by the investigator, were assessed as prespecified exploratory endpoints.

Safety endpoints included treatment-emergent adverse events and serious adverse events that occurred during the reporting period. Major adverse cardiovascular events, acute pancreatitis and deaths were reviewed by an independent external adjudication committee.

### Statistical analysis

A sample size of 600 participants provided power of >90% to demonstrate the superiority of tirzepatide MTD to placebo, for the coprimary endpoints, each at a two-sided significance level of 0.05. Sample size calculation assumed a difference of at least 12% in mean per cent weight reduction from randomization to week 72 for tirzepatide MTD as compared with placebo, a common s.d. of 10% and a dropout rate of 25%. Efficacy and safety endpoints were analyzed with data from all randomly assigned participants who took at least one dose of study drug (modified intention-to-treat population).

Two estimands (TRE and efficacy) were used to assess treatment efficacy from different perspectives and accounted for intercurrent events differently.

The TRE uses a treatment policy strategy to handle intercurrent events (ICH E9(R1)) and is intended to give an estimation of the average treatment effect of tirzepatide relative to placebo for all participants who had undergone randomization, regardless of treatment adherence. For estimation for this estimand, the intercurrent events and resulting missing values were handled by a hybrid approach using retrieved dropouts imputation from the same treatment group or using all nonmissing data assuming missing at random. This estimand is therefore also referred to as a ‘hybrid’ estimand in the study protocol. Continuous endpoints were analyzed using an analysis of covariance model, and categorical endpoints were analyzed by logistical regression. Both models included randomized treatment and stratification factors (country/pooled country, sex and per cent body weight reduction at the end of lead-in (<10 and ≥10%) as fixed effects, and baseline measure as a covariate. Analyses were conducted with hybrid imputation of missing body weight at 72 weeks and statistical inference over hybrid imputation of missing data guided by Rubin^47^.

Specifically, for missing data solely due to COVID-19, missing data were considered as missing at random and imputed using all available nonmissing data of the outcome measurement from the same treatment arm; for missing data due to other intercurrent events, these were imputed based on retrieved dropouts in the same treatment arm, defined as observed primary outcome measurements, from participants in the same treatment group, who had their efficacy assessed after early discontinuation of the study drug.

The efficacy estimand uses a hypothetical strategy to handle intercurrent events (ICH E9(R1)) and represented the average treatment effect of tirzepatide relative to placebo, before treatment discontinuation, for all participants who had undergone randomization. The resulting missing values (unobserved, discarded) after treatment discontinuation were implicitly handled using a mixed model for repeated measures (MMRM) under the assumption of missing at random. Continuous endpoints were analyzed using a MMRM model, and categorical endpoints by logistical regression. MMRM analysis included randomized treatment, visit, treatment-by-visit interaction and stratification factors (country/pooled country, sex and per cent body weight reduction at the end of lead-in (<10 and ≥10%) as fixed effects, and baseline measure as a covariate. The logistical regression model included randomized treatment, the same stratification factors as fixed effects and baseline measure as a covariate. Missing values were imputed by the predicted value from the MMRM model above, and continuous measurements were then dichotomized to binary outcomes. The type 1 error rate was controlled at a level of 0.05 within each estimand for evaluation of primary and key secondary objectives.

Statistical analyses were carried out using SAS v.9.4, unless otherwise specified.

### Reporting summary

Further information on research design is available in the Nature Portfolio Reporting Summary linked to this article.

## Online content

Any methods, additional references, Nature Portfolio reporting summaries, source data, extended data, supplementary information, acknowledgements, peer review information; details of author contributions and competing interests; and statements of data and code availability are available at 10.1038/s41591-023-02597-w.

### Supplementary information

Supplementary Information List of investigators, protocol and statistical analysis plan.

Reporting Summary

## Figures and Tables

**Extended Data Table 1:**
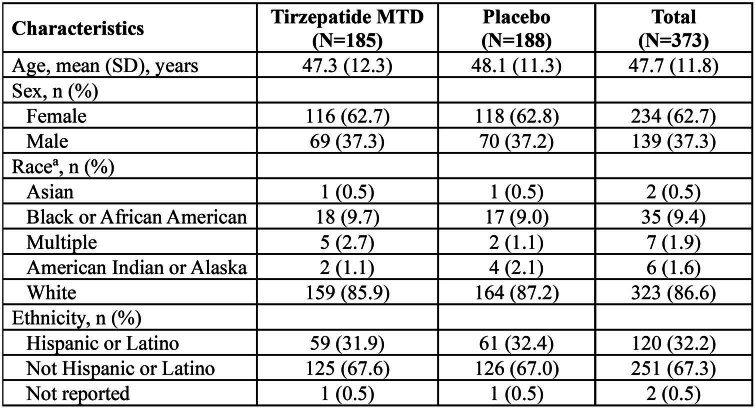
Demographics of Participants in the United States

^a^Race and ethnicity were determined by the participant according to fixed selection categories. MTD, maximum tolerated dose (10 or 15 mg).

**Extended Data Table 2:**
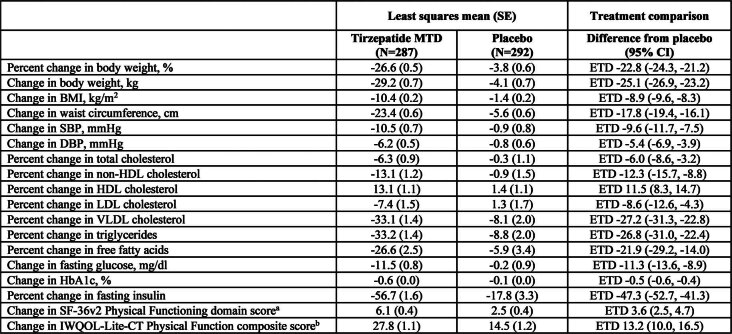
Additional Efficacy Findings from Start of Intensive Lifestyle Intervention Lead-In (Week-12) to Week 72 (Efficacy Estimand)

BMI, body mass index; DBP, diastolic blood pressure; ETD, estimated treatment difference; HbA1c, glycated hemoglobin; HDL-C, high-density lipoprotein cholesterol; IWQOL-Lite-CT, Impact of Weight on Quality of Life-Lite-Clinical Trials Version; LDL-C, low-density lipoprotein cholesterol; MTD, maximum tolerated dose (10 or 15 mg); SBP, systolic blood pressure; SF-36v2, Short Form-36 version 2 Health Survey acute form; VLDL-C, very low-density lipoprotein cholesterol. ^a^The SF-36v2 measures health-related quality of life and general health status. The SF-36v2 scores are norm-based scores, ie, scores transformed to a scale in which the 2009 US general population has a mean score of 50 and an SD of 10. An increase in score represents an improvement in health status. ^b^The IWQOL-Lite-CT measures weight-specific health-related quality of life. All items are rated on either a 5-point frequency scale (‘never’ to ‘always’) or a 5-point truth scale (‘not at all true’ to ‘completely true’). Scores are transformed to a scale of 0 to 100, with higher scores reflecting better levels of functioning.

**Extended Data Table 3:**
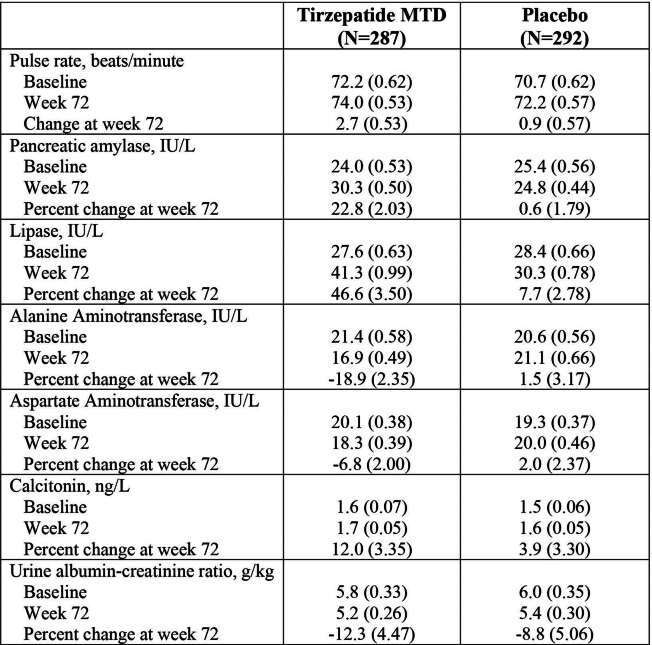
Additional Safety Measures from Randomization to Week 72

Data are model based estimate (standard error) and were analyzed with log transformation, except for pulse rate data. MTD, maximum tolerated dose (10 or 15 mg).

## Data Availability

Eli Lilly and Company provides access to all individual participant data collected during the trial, after anonymization, except for pharmacokinetic or genetic data. Data are available to request 6 months after the indication studied has been approved in the USA and European Union and after primary publication acceptance, whichever is later. No expiration date of data requests is currently set once data have been made available. Access is provided after a proposal has been approved by an independent review committee identified for this purpose and after receipt of a signed data-sharing agreement. Data and documents, including the study protocol, statistical analysis plan, clinical study report and blank or annotated case report forms, will be provided in a secure data-sharing environment. For details on submitting a request, see the instructions provided at www.vivli.org.
