# Author Correction: Tirzepatide after intensive lifestyle intervention in adults with overweight or obesity: the SURMOUNT-3 phase 3 trial

**DOI:** 10.1038/s41591-024-02883-1

**Published:** 2024-02-27

**Authors:** Thomas A. Wadden, Ariana M. Chao, Sriram Machineni, Robert Kushner, Jamy Ard, Gitanjali Srivastava, Bruno Halpern, Shuyu Zhang, Jiaxun Chen, Mathijs C. Bunck, Nadia N. Ahmad, Tammy Forrester

**Affiliations:** 1 Department of Psychiatry, Perelman School of Medicine at the University of Pennsylvania, Philadelphia, PA USA; 2 Johns Hopkins School of Nursing, Baltimore, MD USA; 3 Division of Endocrinology and Metabolism, Department of Medicine, Albert Einstein College of Medicine, Bronx, NY USA; 4 Department of Medicine, Northwestern University Feinberg School of Medicine, Chicago, IL USA; 5 Department of Epidemiology and Prevention, Wake Forest School of Medicine, Winston-Salem, NC USA; 6 Department of Medicine, Division of Diabetes, Endocrinology & Metabolism, Department of Pediatrics, Department of Surgery, Vanderbilt University School of Medicine, Nashville, TN USA; 7 Vanderbilt Weight Loss Clinics, Vanderbilt University Medical Center, Nashville, TN USA; 8 Obesity Group, Department of Endocrinology, Universidade de São Paulo, São Paulo, Brazil; 9 Eli Lilly and Company, Indianapolis, IN USA

**Keywords:** Obesity, Metabolic disorders

Correction to: *Nature Medicine* 10.1038/s41591-023-02597-w, published online 15 October 2023.

In the originally published version of this article, the labels in the Fig. 2 key for panels b and f (now shown as “Tirzepatide MTD” and “Placebo” for the inverted blue triangle and white box, respectively) were swapped. This error has been corrected in the HTML and PDF versions of the article.

