# Supplementary material for: Tirzepatide after intensive lifestyle intervention in adults with overweight or obesity: the SURMOUNT-3 phase 3 trial
Source: Nat Med. 2023 Oct 15;29(11):2909–18. doi: 10.1038/s41591-023-02597-w (PMC10667099; doi:10.1038/s41591-023-02597-w)
Supplement: Supplementary file 2 — Reporting Summary [file 41591_2023_2597_MOESM2_ESM.pdf]

nature portfolio

Corresponding author(s): Thomas Wadden

Last updated by author(s): 13th Sept 2023



# Reporting Summary

Nature Portfolio wishes to improve the reproducibility of the work that we publish. This form provides structure for consistency and transparency in reporting. For further information on Nature Portfolio policies, see our Editorial Policies and the Editorial Policy Checklist.

Please do not complete any field with "not applicable" or n/a. Refer to the help text for what text to use if an item is not relevant to your study.
For final submission: please carefully check your responses for accuracy; you will not be able to make changes later.

## Statistics

For all statistical analyses, confirm that the following items are present in the figure legend, table legend, main text, or Methods section.

| n/a | Confirmed | |
|---|---|---|
| ☐ | ☒ | The exact sample size ($n$) for each experimental group/condition, given as a discrete number and unit of measurement |
| ☒ | ☐ | A statement on whether measurements were taken from distinct samples or whether the same sample was measured repeatedly |
| ☐ | ☒ | The statistical test(s) used AND whether they are one- or two-sided<br>*Only common tests should be described solely by name; describe more complex techniques in the Methods section.* |
| ☐ | ☒ | A description of all covariates tested |
| ☒ | ☐ | A description of any assumptions or corrections, such as tests of normality and adjustment for multiple comparisons |
| ☐ | ☒ | A full description of the statistical parameters including central tendency (e.g. means) or other basic estimates (e.g. regression coefficient) AND variation (e.g. standard deviation) or associated estimates of uncertainty (e.g. confidence intervals) |
| ☐ | ☒ | For null hypothesis testing, the test statistic (e.g. $F$, $t$, $r$) with confidence intervals, effect sizes, degrees of freedom and $P$ value noted<br>*Give P values as exact values whenever suitable.* |
| ☒ | ☐ | For Bayesian analysis, information on the choice of priors and Markov chain Monte Carlo settings |
| ☒ | ☐ | For hierarchical and complex designs, identification of the appropriate level for tests and full reporting of outcomes |
| ☒ | ☐ | Estimates of effect sizes (e.g. Cohen's $d$, Pearson's $r$), indicating how they were calculated |

*Our web collection on statistics for biologists contains articles on many of the points above.*

## Software and code

Policy information about availability of computer code

| | |
|---|---|
| Data collection | No specific software was used for data collection; the collected data were entered using the Veeva Vault Clinical Data Management Suite (CDMS) electronic data collection system. |
| Data analysis | Statistical analyses were done using SAS version 9.4, unless otherwise specified. |

For manuscripts utilizing custom algorithms or software that are central to the research but not yet described in published literature, software must be made available to editors and reviewers. We strongly encourage code deposition in a community repository (e.g. GitHub). See the Nature Portfolio guidelines for submitting code & software for further information.

## Data

Policy information about availability of data

All manuscripts must include a data availability statement. This statement should provide the following information, where applicable:
- Accession codes, unique identifiers, or web links for publicly available datasets
- A description of any restrictions on data availability
- For clinical datasets or third party data, please ensure that the statement adheres to our policy

Lilly provides access to all individual participant data collected during the trial, after anonymization, except for pharmacokinetic or genetic data. Data are available to request 6 months after the indication studied has been approved in the US and EU and after primary publication acceptance, whichever is later. No expiration date of data requests is currently set once data are made available. Access is provided after a proposal has been approved by an independent review committee identified for this purpose and after receipt of a signed data sharing agreement. Data and documents, including the study protocol, statistical analysis plan, clinical study report, blank or annotated case report forms, will be provided in a secure data sharing environment. For details on submitting a request, see the instructions provided at www.vivli.org.

The protocol and statistical analysis plan are included in the supplementary material.

## Research involving human participants, their data, or biological material

Policy information about studies with human participants or human data. See also policy information about sex, gender (identity/presentation), and sexual orientation and race, ethnicity and racism.

| | |
|---|---|
| Reporting on sex and gender | Patients were screened and enrolled irrespective of their sex. Female enrollment was capped at 70% to ensure adequate representation of the male population. Any data on sex was collected at each clinical trial site. Data are provided in Table 2 and Extended Data Table 1. |
| Reporting on race, ethnicity, or other socially relevant groupings | Patients were screened and enrolled irrespective of their race/ethnicity. Data are provided in Table 2 and Extended Data Table 1. |
| Population characteristics | Provided in Table 2 and Extended Data Table 1. |
| Recruitment | Took place in medical research centers in the United States, Argentina, and Brazil. |
| Ethics oversight | The protocol was approved by local institutional review boards, and the trial complied with the International Conference on Harmonization Good Clinical Practice guidelines and the Declaration of Helsinki. The ethics review boards were Comité de Ética Independiente Consultorios Integrados CEICI, COMITE DE ETICA EN INVESTIGACION CLINICA CEIC, COMITÉ DE ÉTICA SAAVEDRA, Centro de Investigaciones Metabólicas (CINME), CEMEDIAB, INVESTIGA - INSTITUTO DE PESQUISA, Comitê de Ética em Pesquisa em Seres Humanos do Hospital Pró-Cardíaco, Comitê de Ética em Pesquisa Faculdade de Medicina do ABC, Comitê de Ética em Pesquisa em Seres Humanos - Centro Integrado de Atenção à Saúde - CIAS – UNIMED, Instituto de Saúde e Bem-Estar da Mulher (ISBEM), Comitê de Ética em Pesquisa em Seres Humanos HC-UFPR, Instituto de Saúde e Bem-Estar da Mulher (ISBEM), Universidade Positivo, Advarra Inc., and Vanderbilt Human Research Protections Program. |

Note that full information on the approval of the study protocol must also be provided in the manuscript.

# Field-specific reporting

Please select the one below that is the best fit for your research. If you are not sure, read the appropriate sections before making your selection.

[X] Life sciences [ ] Behavioural & social sciences [ ] Ecological, evolutionary & environmental sciences

For a reference copy of the document with all sections, see nature.com/documents/nr-reporting-summary-flat.pdf

# Life sciences study design

All studies must disclose on these points even when the disclosure is negative.

| | |
|---|---|
| Sample size | The sample size was determined based on achieving approximately 600 participants randomly assigned to study drug intervention (300 participants per treatment group). Sample size determination assumed that evaluation of superiority of tirzepatide MTD (10 mg or 15 mg) to placebo would be conducted at a 2-sided significance level of 0.05 using a 2-sample t-test. Additionally, a difference of at least 12% mean body weight percentage reduction from randomization at 72 weeks for tirzepatide MTD compared to placebo, a common SD of 10%, and a dropout rate of 25% were assumed for statistical power calculations. Under the assumptions above, randomizing 600 participants in a 1:1 ratio to MTD (300 participants) and placebo (300 participants) provided more than 90% power to demonstrate superiority of tirzepatide MTD to placebo.The chosen sample size and randomization ratio also provided >90% power to establish superiority of tirzepatide MTD to placebo in terms of proportion of participants achieving at least 5% body weight reduction from randomization at 72 weeks, based on a Chi-square test at a 2-sided significance level of 0.05, assuming 20% placebo treated participants and 46% tirzepatide treated participants achieved the goal and a dropout rate of 25%. |
| Data exclusions | In this study, all randomly assigned participants took at least one dose of study intervention. Therefore, the intention-to-treat population is the same as the modified intention-to-treat population. Two estimands, (treatment-regimen estimand and efficacy estimand), were used to assess treatment efficacy from different perspectives and accounted for intercurrent events differently. The treatment-regimen estimand represented the average treatment effect of tirzepatide relative to placebo for all participants who had undergone randomization, regardless of treatment adherence.The efficacy estimand represented the average treatment effect of tirzepatide relative to placebo, prior to treatment discontinuation, for all participants who had undergone randomization. |
| Replication | Results were consistent between the protocol defined estimands. This study is part of a broader phase 3 program (SURMOUNT). |
| Randomization | Participants who achieved ≥5.0% weight reduction at the end of the 12-week lead-in period were randomly assigned in a 1:1 ratio to receive either the maximum tolerated dose (MTD) of tirzepatide (10 mg or 15 mg) or placebo. Assignment to treatment group was determined by a computer-generated random sequence using a validated interactive web-response system. Randomization was stratified according to country, sex (female, male), and percent weight reduction at the end of lead-in (<10% versus ≥10%). |
| Blinding | All participants, investigators, and the sponsor were masked to treatment assignment. To maintain masking of participants and site staff, the single-dose pens were identical between active product and placebo. |

# Behavioural & social sciences study design

All studies must disclose on these points even when the disclosure is negative.

| | |
|---|---|
| Study description | |
| Research sample | |
| Sampling strategy | |
| Data collection | |
| Timing | |
| Data exclusions | |
| Non-participation | |
| Randomization | |

# Ecological, evolutionary & environmental sciences study design

All studies must disclose on these points even when the disclosure is negative.

| | |
|---|---|
| Study description | |
| Research sample | |
| Sampling strategy | |
| Data collection | |
| Timing and spatial scale | |
| Data exclusions | |
| Reproducibility | |
| Randomization | |
| Blinding | |

Did the study involve field work? ☐ Yes ☐ No

## Field work, collection and transport

| | |
|---|---|
| Field conditions | |
| Location | |
| Access & import/export | |
| Disturbance | |

# Reporting for specific materials, systems and methods

We require information from authors about some types of materials, experimental systems and methods used in many studies. Here, indicate whether each material, system or method listed is relevant to your study. If you are not sure if a list item applies to your research, read the appropriate section before selecting a response.

### Materials & experimental systems

| n/a | Involved in the study | |
|---|---|---|
| X | | Antibodies |
| X | | Eukaryotic cell lines |
| X | | Palaeontology and archaeology |
| X | | Animals and other organisms |
| | X | Clinical data |
| X | | Dual use research of concern |
| X | | Plants |

### Methods

| n/a | Involved in the study | |
|---|---|---|
| X | | ChIP-seq |
| X | | Flow cytometry |
| X | | MRI-based neuroimaging |

## Antibodies

| | |
|---|---|
| Antibodies used | |
| Validation | |

# Eukaryotic cell lines

Policy information about cell lines and Sex and Gender in Research

Cell line source(s)

Authentication

Mycoplasma contamination

Commonly misidentified lines
(See ICLAC register)

# Palaeontology and Archaeology

Specimen provenance

Specimen deposition

Dating methods

☐ Tick this box to confirm that the raw and calibrated dates are available in the paper or in Supplementary Information.

Ethics oversight

Note that full information on the approval of the study protocol must also be provided in the manuscript.

# Animals and other research organisms

Policy information about studies involving animals; ARRIVE guidelines recommended for reporting animal research, and Sex and Gender in Research

Laboratory animals

Wild animals

Reporting on sex

Field-collected samples

Ethics oversight

Note that full information on the approval of the study protocol must also be provided in the manuscript.

# Clinical data

Policy information about clinical studies
All manuscripts should comply with the ICMJE guidelines for publication of clinical research and a completed CONSORT checklist must be included with all submissions.

Clinical trial registration: ClinicalTrials.gov, NCT04657016

Study protocol: Provided in the supplementary material

Data collection: Data were collected at 62 individual medical research centres in the United States, Argentina, and Brazil. The first participant was enrolled on 12th April 2021 and the last participant on 3rd September 2021, with the last participant's last visit occurring on 12th May 2023.

Outcomes: Primary and secondary objectives and endpoints were pre-specified in the protocol and statistical analysis plan. Co-primary endpoints were the percent change in body weight and the proportion of study participants who achieved ≥5% weight reduction from randomization to week 72. Key secondary endpoints controlled for type 1 error rate included the proportion of study participants who achieved ≥10%, ≥15%, or ≥20% weight reductions from randomization to week 72, the proportion of participants who, at week 72, maintained ≥80% of the body weight loss achieved during the 12-week lead-in period, and the change in waist circumference (cm) from randomization to week 72.

# Dual use research of concern

Policy information about dual use research of concern

## Hazards

Could the accidental, deliberate or reckless misuse of agents or technologies generated in the work, or the application of information presented in the manuscript, pose a threat to:

No | Yes

- [ ] [ ] Public health
- [ ] [ ] National security
- [ ] [ ] Crops and/or livestock
- [ ] [ ] Ecosystems
- [ ] [ ] Any other significant area

## Experiments of concern

Does the work involve any of these experiments of concern:

No | Yes

- [ ] [ ] Demonstrate how to render a vaccine ineffective
- [ ] [ ] Confer resistance to therapeutically useful antibiotics or antiviral agents
- [ ] [ ] Enhance the virulence of a pathogen or render a nonpathogen virulent
- [ ] [ ] Increase transmissibility of a pathogen
- [ ] [ ] Alter the host range of a pathogen
- [ ] [ ] Enable evasion of diagnostic/detection modalities
- [ ] [ ] Enable the weaponization of a biological agent or toxin
- [ ] [ ] Any other potentially harmful combination of experiments and agents

# Plants

Seed stocks

Novel plant genotypes

Authentication

# ChIP-seq

## Data deposition

- [ ] Confirm that both raw and final processed data have been deposited in a public database such as GEO.
- [ ] Confirm that you have deposited or provided access to graph files (e.g. BED files) for the called peaks.

Data access links
*May remain private before publication.*

Files in database submission

Genome browser session
(e.g. UCSC)

## Methodology

Replicates

Sequencing depth

Antibodies

Peak calling parameters

Data quality

Software

# Flow Cytometry

## Plots

Confirm that:

- [ ] The axis labels state the marker and fluorochrome used (e.g. CD4-FITC).
- [ ] The axis scales are clearly visible. Include numbers along axes only for bottom left plot of group (a 'group' is an analysis of identical markers).
- [ ] All plots are contour plots with outliers or pseudocolor plots.
- [ ] A numerical value for number of cells or percentage (with statistics) is provided.

## Methodology

Sample preparation

Instrument

Software

Cell population abundance

Gating strategy

- [ ] Tick this box to confirm that a figure exemplifying the gating strategy is provided in the Supplementary Information.

# Magnetic resonance imaging

## Experimental design

Design type

Design specifications

Behavioral performance measures

Imaging type(s)

Field strength

Sequence & imaging parameters

Area of acquisition

Diffusion MRI ☐ Used ☐ Not used

## Preprocessing

Preprocessing software

Normalization

Normalization template

Noise and artifact removal

Volume censoring

## Statistical modeling & inference

Model type and settings

Effect(s) tested

Specify type of analysis: ☐ Whole brain ☐ ROI-based ☐ Both

Statistic type for inference

(See Eklund et al. 2016)

Correction

## Models & analysis

| n/a | Involved in the study |
|---|---|
| ☐ | ☐ Functional and/or effective connectivity |
| ☐ | ☐ Graph analysis |
| ☐ | ☐ Multivariate modeling or predictive analysis |

Functional and/or effective connectivity

Graph analysis

Multivariate modeling and predictive analysis





This checklist template is licensed under a Creative Commons Attribution 4.0 International License, which permits use, sharing, adaptation, distribution and reproduction in any medium or format, as long as you give appropriate credit to the original author(s) and the source, provide a link to the Creative Commons license, and indicate if changes were made. The images or other third party material in this article are included in the article's Creative Commons license, unless indicated otherwise in a credit line to the material. If material is not included in the article's Creative Commons license and your intended use is not permitted by statutory regulation or exceeds the permitted use, you will need to obtain permission directly from the copyright holder. To view a copy of this license, visit http://creativecommons.org/licenses/by/4.0/
